# Ammonia Metabolism in the Aging Liver: Emerging Mechanistic Insights

**DOI:** 10.1111/acel.70639

**Published:** 2026-07-21

**Authors:** Heng Zhang, Guangyu Liang, Anding Liu

**Affiliations:** ^1^ Experimental Medicine Center, Tongji Hospital, Tongji Medical College Huazhong University of Science and Technology Wuhan China; ^2^ Key Laboratory of Vascular Aging, Ministry of Education, Tongji Hospital of Tongji Medical College Huazhong University of Science and Technology Wuhan China

**Keywords:** aging, ammonia, liver

## Abstract

During aging, hepatic structural, metabolic, and regulatory impairments collectively contribute to the decline of hepatic and systemic function. As a core hepatic physiological process, ammonia metabolism is essential for maintaining systemic nitrogen homeostasis. However, how ammonia metabolism is altered during aging, and whether these changes contribute to hepatic and systemic decline, remain insufficiently understood. In this review, current evidence linking hepatic ammonia metabolism to liver aging is summarized. The major pathways of hepatic ammonia disposal, including the urea cycle and glutamine synthesis, are first outlined. Age‐related changes in these pathways are then discussed, with emphasis on mitochondrial dysfunction, altered post‐translational regulation, transcriptional and epigenetic remodeling, and disruption of metabolic zonation. Emerging evidence that ammonia functions not only as a nitrogen waste product but also as a bioactive stress signal is also reviewed. In this context, ammonia has been implicated in mitochondrial injury, senescence‐associated signaling, proteostasis defects, and inflammatory and fibrogenic remodeling. The systemic consequences of ammonia dysregulation are further considered, particularly along the liver–brain, liver–muscle, and liver–gut axes. Finally, current and emerging therapeutic strategies are evaluated, including ammonia‐lowering agents, senotherapeutics, and microbiota‐directed approaches. Collectively, this review identify ammonia metabolism as an underappreciated but potentially axis for understanding liver aging, thereby providing a framework for future mechanistic and translational studies.

## Graphical abstract

Liver aging may weaken ammonia‐detoxification reserve by disrupting mitochondrial ureagenesis, GS‐mediated scavenging, glutamine metabolism, and metabolic zonation. This emerging framework links impaired nitrogen handling to potential hepatic stress amplification and systemic crosstalk, while highlighting the need for direct validation in physiological aging.

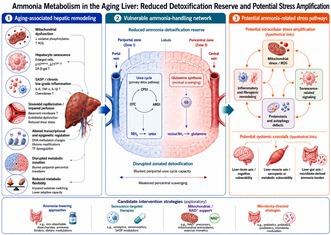

## 1 Introduction

Aging is a complex biological process characterized by a progressive decline in physiological function and disruption of metabolic homeostasis, which increases susceptibility to chronic multi‐organ pathologies (Kennedy et al. 2014; Lopez‐Otin et al. 2023). Among the organs involved in metabolic regulation, the liver plays a central role in maintaining systemic metabolic balance. As the primary organ responsible for nutrient processing, detoxification, and nitrogen metabolism, the liver coordinates multiple metabolic pathways that are essential for organismal homeostasis (Steinberg et al. 2025). However, the aging process induces profound structural and functional remodeling of the liver, including hepatocyte senescence, alterations in hepatic microarchitecture, and reduced regenerative capacity (Ganguly et al. 2025; Maeso‐Diaz et al. 2018). These changes are accompanied by substantial metabolic remodeling, which may affect several key metabolic pathways. While research has extensively focused on age‐related shifts in glucose and lipid metabolism, the impact of aging on nitrogen homeostasis, particularly ammonia detoxification, remains relatively under‐explored despite its critical importance for systemic stability.

Ammonia is a nitrogen‐containing compound generated mainly from amino acid catabolism, intestinal microbial activity, and nucleotide metabolism (Jakhar et al. 2024). Due to the toxicity of elevated ammonia levels, particularly to the central nervous system, the concentration of ammonia must be strictly regulated (Gallego‐Duran et al. 2024; Sarin Zacharia and Jacob 2024). The liver maintains systemic ammonia homeostasis primarily through two principal pathways: the urea cycle in periportal hepatocytes and glutamine synthesis in perivenous hepatocytes (Ben‐Moshe and Itzkovitz 2019). These processes operate in a spatially coordinated manner within the liver lobule, thereby ensuring efficient ammonia clearance and preventing systemic accumulation.

While direct experimental evidence remains limited, it is hypothesized that aging may influence this delicate homeostatic balance of hepatic ammonia metabolism. Conditions such as mitochondrial dysfunction, oxidative stress, and metabolic remodeling are frequently observed in general aging and related diseases like MASLD (Zhao et al. 2023). Consequently, these age‐associated declines are expected to theoretically impair the efficiency of hepatic ammonia detoxification. In addition, aging is often accompanied by disruptions in hepatic metabolic zonation (Sinha et al. 2025), which may further compromise the coordinated handling of nitrogen metabolites. Although severe hyperammonemia is typically associated with advanced liver diseases, subtle disturbances in ammonia metabolism may also occur during physiological aging and contribute to metabolic imbalance.

Beyond its traditional role as a metabolic waste product, ammonia has recently been recognized as a biologically active molecule that can influence cellular signaling and metabolic regulation (Njei et al. 2024). Accumulating evidence indicates that elevated ammonia levels can induce oxidative stress, mitochondrial dysfunction, and cellular senescence in extrahepatic cells (Kumar et al. 2021). In the context of liver aging, these processes may establish a feedback loop in which impaired ammonia metabolism exacerbates cellular stress and further accelerates the decline of hepatic function. However, the direct relationship between ammonia dysmetabolism and physiological aging is still in its infancy and largely unproven, as concrete experimental data establishing a direct causal link remain lacking at this stage.

This review provides a comprehensive synthesis of contemporary insights regarding the potential mechanistic connections between ammonia metabolism and hepatic senescence. An overview of the principal pathways involved in hepatic ammonia detoxification is first presented. Subsequently, age‐related changes in hepatic structure and metabolism that may influence nitrogen handling are discussed. Particular attention is given to the molecular mechanisms that connect ammonia metabolism with mitochondrial dysfunction, oxidative stress, and cellular senescence. Furthermore, the systemic ramifications of these metabolic alterations are evaluated. Finally, emerging perspectives on the modulation of ammonia metabolism as a potential strategy to mitigate metabolic decline associated with aging are highlighted.

## 2 Hepatic Ammonia Metabolism

Ammonia metabolism is a central component of nitrogen homeostasis in mammals. Because ammonia is continuously generated during normal metabolic processes, efficient detoxification mechanisms are required to maintain its concentration within a safe physiological range. The liver plays the dominant role in systemic ammonia clearance through coordinated metabolic pathways that convert ammonia into less toxic nitrogen‐containing compounds (Adeva et al. 2012; Haussinger and Schliess 2008). These pathways operate in a highly organized manner within hepatocytes and rely on the integration of mitochondrial metabolism, enzymatic activity, and spatial compartmentalization within the hepatic lobule (Ben‐Moshe and Itzkovitz 2019).

### 2.1 Sources of Ammonia

Ammonia is produced from several endogenous and exogenous sources in the body. One major source is the catabolism of amino acids during protein metabolism. Deamination reactions occurring in peripheral tissues release ammonia as a byproduct of amino acid breakdown, which is subsequently transported through the bloodstream to the liver for detoxification (Wu 2009).

The gastrointestinal tract represents another important source of systemic ammonia. Intestinal microbiota generate substantial amounts of ammonia through the metabolism of dietary proteins and nitrogen‐containing compounds. Urease‐producing bacteria in particular contribute to the hydrolysis of urea into ammonia and carbon dioxide (Davila et al. 2013). A portion of the ammonia produced in the gut enters the portal circulation and is delivered directly to the liver, where it is rapidly metabolized under normal physiological conditions.

Additional sources of ammonia include nucleotide metabolism and other cellular biochemical reactions. During purine and pyrimidine degradation, nitrogen atoms are released in the form of ammonia (Lane and Fan 2015). Furthermore, metabolic processes such as glutamine deamidation and transamination reactions can also contribute to intracellular ammonia production (Cruzat et al. 2018). Although these reactions are essential for normal cellular metabolism, they require efficient detoxification systems to prevent toxic accumulation.

In addition to the systemic influx of gut‐derived ammonia, the intrahepatic hydrolysis of glutamine by glutaminase enzymes represents a major endogenous source of ammonia within the liver. The liver utilizes two distinct glutaminase isoforms, specifically the liver‐type (GLS2) and kidney‐type (GLS1) variants, which are subject to precise spatial and metabolic regulation (Mates et al. 2013). Under physiological conditions, GLS2 is predominantly localized within periportal hepatocytes, where it catalyzes the conversion of glutamine into glutamate and ammonia (Haussinger 1990). This mitochondrial reaction is uniquely characterized by its positive activation by ammonia itself, creating a feed‐forward mechanism that links glutamine breakdown directly to the urea cycle for subsequent nitrogen clearance (Haussinger 1990; Mates et al. 2013). In contrast, while parenchymal GLS1 expression remains low in the healthy liver, its induction during tissue stress, metabolic overloading, or cellular senescence can drastically accelerate localized ammonia generation. Consequently, hepatic glutaminase activity serves as a primary intrahepatic source of ammonia, tightly coupling amino acid catabolism with the dynamic maintenance of systemic nitrogen homeostasis.

Because ammonia can readily cross biological membranes and disrupt cellular processes, maintaining systemic ammonia homeostasis is critical for organismal health (Braissant et al. 2013; Rehman et al. 2025). The liver therefore functions as the primary organ responsible for ammonia detoxification, converting ammonia into less toxic compounds that can be safely excreted or further metabolized.

### 2.2 Ammonia Detoxification Pathways in the Liver

Hepatic ammonia detoxification is mainly achieved through two complementary metabolic pathways: the urea cycle and glutamine synthesis. These pathways operate sequentially within different populations of hepatocytes and together ensure efficient removal of ammonia from the circulation (Adeva et al. 2012).

#### 2.2.1 The Urea Cycle

The urea cycle is the primary pathway responsible for ammonia detoxification in the liver. This metabolic process converts ammonia into urea, which can then be transported through the bloodstream to the kidneys and excreted in urine (Shou et al. 2025). The urea cycle involves a series of enzymatic reactions that occur partly in the mitochondrial matrix and partly in the cytosol of hepatocytes (Caldwell et al. 2018).

The first and rate‐limiting step of the urea cycle is catalyzed by carbamoyl phosphate synthetase 1 (CPS1), which converts ammonia and bicarbonate into carbamoyl phosphate using ATP. This reaction takes place within mitochondria and represents a key regulatory point of nitrogen metabolism (Nitzahn and Lipshutz 2020). Carbamoyl phosphate is subsequently converted into citrulline through the action of ornithine transcarbamylase. Citrulline then exits the mitochondria and enters the cytosol, where it participates in additional enzymatic reactions involving argininosuccinate synthetase, argininosuccinate lyase, and arginase (Shou et al. 2025).

The final product of this pathway is urea, which is released into the bloodstream for renal excretion. The urea cycle is an energy‐dependent process that requires adequate mitochondrial function and ATP supply (Natesan et al. 2016). Consequently, any disruption of mitochondrial metabolism may influence the efficiency of ammonia detoxification in hepatocytes.

#### 2.2.2 Glutamine Synthesis

In addition to the urea cycle, ammonia can be detoxified through the synthesis of glutamine from glutamate. This reaction is catalyzed by glutamine synthetase (GS) and represents an important secondary pathway for ammonia removal (Hakvoort et al. 2017). Glutamine produced in hepatocytes can serve as a nitrogen carrier and may be transported to other tissues where it participates in various metabolic processes (Yoo et al. 2020; Zhu et al. 2022).

GS plays a particularly important role in capturing residual ammonia that escapes the urea cycle. By converting ammonia into glutamine, this pathway helps prevent the accumulation of free ammonia in the circulation and contributes to maintaining nitrogen balance in the body (Castegna and Menga 2018).

### 2.3 Metabolic Zonation in Hepatic Ammonia Metabolism

A distinctive feature of hepatic ammonia metabolism is its spatial organization within the liver lobule, commonly referred to as metabolic zonation (Halpern et al. 2017). Hepatocytes located in different regions of the liver lobule perform specialized metabolic functions depending on their proximity to the portal vein or central vein.

Periportal hepatocytes, which are located near the portal triad, exhibit high expression of urea cycle enzymes and are primarily responsible for the initial detoxification of ammonia entering the liver through the portal circulation (Moor et al. 2018). These cells efficiently convert ammonia into urea, thereby removing the majority of circulating ammonia.

In contrast, perivenous hepatocytes located near the central vein express high levels of glutamine synthetase. These cells function as a secondary ammonia‐scavenging system by converting residual ammonia into glutamine (Aizarani et al. 2019). This sequential arrangement of metabolic pathways ensures highly efficient ammonia clearance and prevents ammonia leakage into the systemic circulation.

The coordinated interaction between the urea cycle and glutamine synthesis pathways, together with the spatial organization of hepatocytes within the liver lobule, provides a robust mechanism for maintaining systemic nitrogen homeostasis (Ben‐Moshe et al. 2019). Disruption of this metabolic organization can compromise ammonia detoxification and contribute to metabolic imbalance.

## 3 Aging‐Associated Changes in Liver Structure and Metabolism

Aging is accompanied by progressive structural and functional alterations in the liver that influence metabolic homeostasis. Although the liver maintains considerable regenerative capacity throughout life, aging gradually impairs hepatocyte turnover, metabolic flexibility, and stress resistance (Le Couteur et al. 2002). These changes can collectively affect multiple metabolic pathways, including lipid metabolism, glucose regulation, and nitrogen disposal (Du et al. 2024). As ammonia detoxification is highly dependent on hepatocyte metabolic capacity and mitochondrial function, age‐associated hepatic remodeling may have important consequences for systemic ammonia metabolism.

### 3.1 Structural Changes in the Aging Liver

Morphological alterations represent one of the most characteristic features of liver aging. Studies have shown that aging is associated with hepatocyte enlargement, accumulation of lipofuscin, and increased cellular heterogeneity within the liver parenchyma (Ogrodnik et al. 2017). In addition, age‐related changes in the hepatic sinusoidal system have been widely reported. These include thickening of the sinusoidal endothelium, loss of fenestrations, and deposition of extracellular matrix components, a process commonly referred to as “pseudocapillarization” of the liver sinusoid (Childs et al. 2015).

Such structural changes can impair the exchange of metabolites between hepatocytes and the circulation, thereby influencing hepatic metabolic efficiency. Reduced sinusoidal permeability may limit substrate delivery and waste removal, potentially affecting metabolic pathways that rely on rapid substrate flux, including nitrogen metabolism (Wiley and Campisi 2021).

Another important feature of liver aging is the decline in regenerative capacity. Although hepatocytes retain the ability to proliferate in response to injury, aging is associated with delayed cell cycle progression and increased cellular senescence. Senescent hepatocytes exhibit altered metabolic activity and increased production of inflammatory mediators, collectively contributing to the development of an age‐associated pro‐inflammatory microenvironment in the liver (Franceschi et al. 2018).

### 3.2 Hepatocyte Senescence and Metabolic Reprogramming

Cellular senescence is increasingly recognized as a key driver of tissue aging. Senescent cells accumulate progressively in multiple organs during aging, including the liver (Sanfeliu‐Redondo et al. 2024). In hepatocytes, senescence is characterized by permanent cell cycle arrest, activation of stress response pathways, and the secretion of pro‐inflammatory factors known as the senescence‐associated secretory phenotype (SASP) (Xing and Zhu 2025).

The presence of senescent hepatocytes can profoundly influence hepatic metabolism. Senescence‐associated metabolic changes include alterations in mitochondrial function, redox balance, and energy metabolism (Zheng et al. 2023). These changes may compromise the ability of hepatocytes to perform metabolically demanding processes such as ammonia detoxification.

Furthermore, senescence‐associated inflammation can influence the hepatic microenvironment by promoting immune cell recruitment and altering intercellular communication. Chronic low‐grade inflammation, commonly referred to as “inflammaging,” has been linked to metabolic dysfunction in aging tissues (Bevilacqua et al. 2023). In the liver, such inflammatory signaling may disrupt the coordination between different hepatocyte populations that normally cooperate to maintain metabolic homeostasis.

### 3.3 Mitochondrial Dysfunction in the Aging Liver

Mitochondria play a central role in hepatic metabolism, serving as key sites for oxidative phosphorylation, fatty acid oxidation, and several steps of the urea cycle. Aging is associated with progressive mitochondrial dysfunction, including reduced respiratory capacity, increased production of reactive oxygen species (ROS), and impaired mitochondrial quality control mechanisms (Deng et al. 2024).

Mitochondrial dysfunction can directly influence nitrogen metabolism because the first step of the urea cycle, catalyzed by CPS1, occurs within the mitochondrial matrix (Zhang et al. 2021). Impairment of mitochondrial energy metabolism may therefore reduce the efficiency of ammonia detoxification in hepatocytes.

In addition, increased oxidative stress associated with mitochondrial dysfunction can damage mitochondrial DNA, proteins, and membranes. Such damage further exacerbates mitochondrial decline and contributes to a vicious cycle of metabolic dysfunction and cellular stress (Zhao et al. 2023). These processes are increasingly recognized as central mechanisms underlying age‐related metabolic disorders.

### 3.4 Alterations in Hepatic Metabolic Zonation During Aging

The liver exhibits a highly organized metabolic architecture known as metabolic zonation, in which hepatocytes located in different regions of the liver lobule perform distinct metabolic functions. This spatial organization is essential for efficient nitrogen metabolism because periportal hepatocytes preferentially express urea cycle enzymes, whereas perivenous hepatocytes predominantly express glutamine synthetase (Paris and Henderson 2022).

Recent studies using single‐cell transcriptomics have revealed that aging can disrupt this spatial metabolic organization. Changes in gene expression patterns across the liver lobule may alter the balance between periportal and perivenous metabolic functions (Nikopoulou et al. 2023). Such alterations may influence the coordinated detoxification of ammonia and reduce the overall efficiency of hepatic nitrogen metabolism.

Moreover, aging‐related changes in signaling pathways, including Wnt/β‐catenin signaling, may contribute to the disruption of metabolic zonation in the liver (Gebhardt et al. 2007). Since this pathway plays a crucial role in maintaining the perivenous hepatocyte identity and glutamine synthetase expression, dysregulation of zonation signaling could potentially impair the secondary ammonia‐scavenging system of the liver.

Collectively, these structural and metabolic alterations suggest that liver aging may significantly influence ammonia metabolism. Although severe hyperammonemia is most commonly associated with advanced liver diseases, subtle age‐related changes in hepatic metabolic capacity may predispose individuals to impaired nitrogen handling. Understanding how aging affects these processes may therefore provide new insights into the metabolic vulnerabilities associated with aging and identify potential targets for therapeutic intervention.

## 4 Alterations in Hepatic Ammonia Metabolism During Aging

**FIGURE 1:**
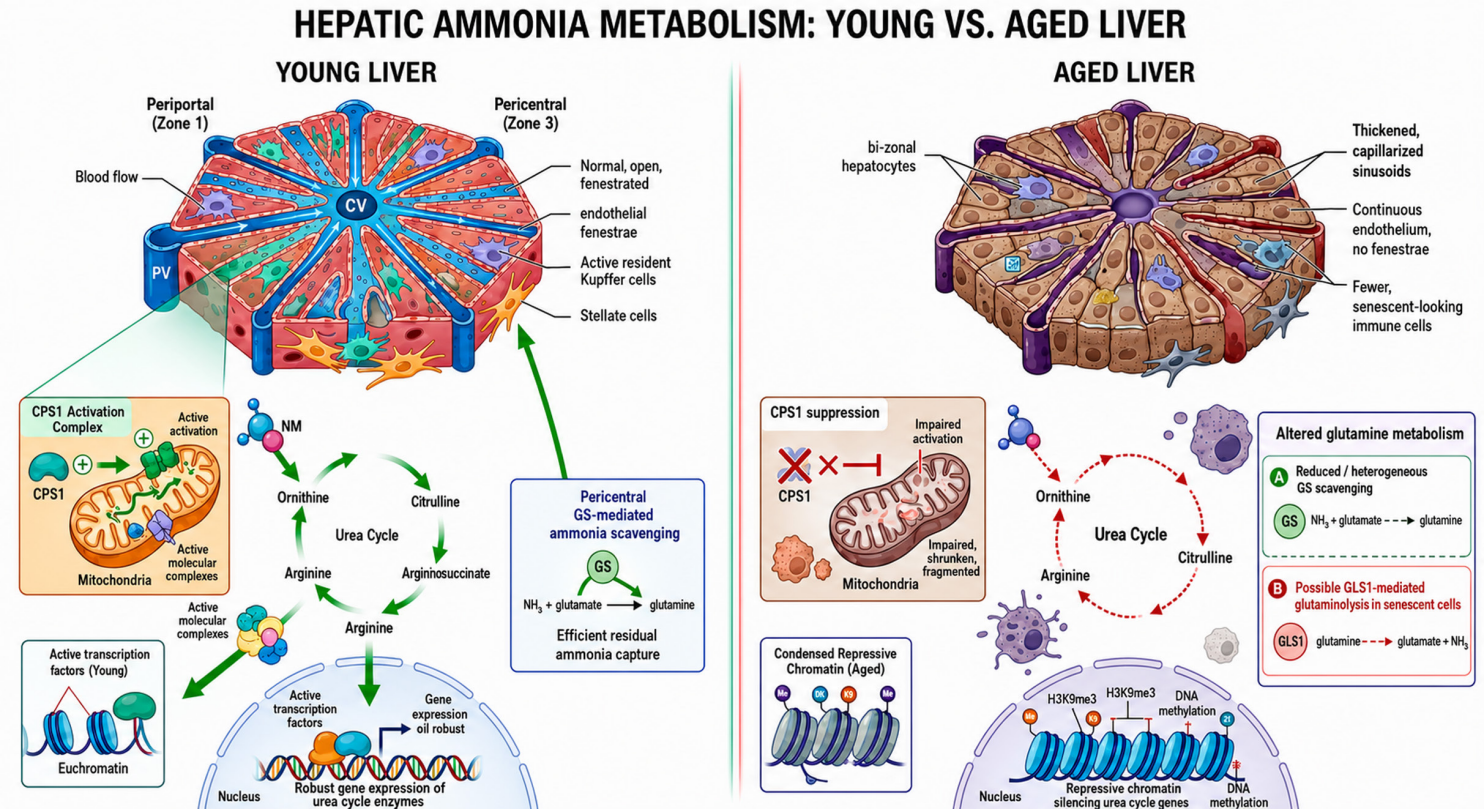
Hepatic ammonia metabolism and zonation in the young and aging liver. The left panel depicts a healthy young liver lobule with intact metabolic zonation, where ammonia is efficiently cleared through a coordinated periportal urea cycle and pericentral glutamine synthesis. The right panel illustrates the aged liver, characterized by sinusoidal pseudocapillarization, loss of strict zonation, and the emergence of aberrant bizonal hepatocytes. Molecular alterations, including CPS1 inhibition and epigenetic repression, collectively impair ammonia detoxification capacity, resulting in elevated systemic ammonia leakage.

It is critical to acknowledge that the precise alterations and underlying mechanisms of hepatic ammonia metabolism during physiological aging remain largely unmapped and lack direct experimental validation. Therefore, the following discussion serves primarily to synthesize the theoretically plausible changes in ammonia‐related molecules, enzymes, and spatial pathways during aging. These potential modifications, inferred from broader age‐associated metabolic shifts, provide a conceptual framework that awaits rigorous future empirical investigation (Figure 1).

### 4.1 Changes in Urea Cycle and Glutamine Metabolism

Aging is accompanied by a progressive decline in hepatic metabolic and detoxification capacity, driven by reduced hepatic perfusion, mitochondrial dysfunction, and alterations in enzyme activity (Le Couteur et al. 2025; Zhong et al. 2025). These changes are expected to influence nitrogen metabolism and ammonia detoxification. However, direct evidence demonstrating impaired ammonia handling under physiological aging conditions remains limited, highlighting a significant gap in current understanding. Given that ammonia clearance in the liver primarily depends on the coordinated activity of the urea cycle and GS pathway, elucidating how these pathways are altered during aging is critical for understanding age‐related disruption of nitrogen homeostasis.

#### 4.1.1 The Urea Cycle

As the primary pathway for hepatic ammonia clearance, the urea cycle is essential for maintaining systemic nitrogen homeostasis. However, while the basic mechanisms of this cycle are well understood, direct experimental evidence regarding how its overall activity and regulatory networks change during physiological aging remains limited. Current research into age‐related urea cycle dysfunction is still in its early stages. Much of our existing knowledge is based on extrapolations from advanced liver diseases or theoretical models of general mitochondrial decline. Therefore, direct experimental validation in the context of healthy aging is highly needed for future studies.

Available multi‐omics studies suggest that aging is accompanied by a broader decline in metabolic protein homeostasis rather than selective suppression of urea cycle genes (Ori et al. 2015). Integrated transcriptomic and proteomic analyses in aged liver indicate a global reduction in metabolic enzyme abundance at the protein level, although these changes are not specific to the urea cycle and are often not paralleled by consistent transcriptional downregulation.

Building upon these age‐related metabolic changes, emerging research highlights distinct expression and functional alterations in specific enzymes within the urea cycle. For instance, evidence from rodent models shows that both the protein abundance and enzymatic activity of Arginase 1 (ARG1) progressively decline with age, despite a compensatory increase in its mRNA levels (Majaw and Sharma 2015). Furthermore, while the total protein expression of the rate‐limiting enzyme CPS1 may remain stable, its functional capacity is significantly suppressed in the aged liver due to increased post‐translational O‐GlcNAcylation (Wu et al. 2022). To advance this field, future investigations should prioritize validating these enzymatic changes in human cohorts and exploring the precise regulatory networks that govern urea cycle dynamics during healthy aging.

Importantly, the structural and functional deficits of these urea cycle enzymes are heavily mirrored—and often accelerated—in Metabolic Dysfunction‐Associated Steatotic Liver Disease (MASLD). As a highly common chronic liver disease driven by metabolic dysfunction, MASLD presents a prominent burden of cellular senescence. Evidence from both animal and human MASLD cohorts indicates that senescent hepatocytes exhibit profound metabolic reprogramming and epigenetic remodeling, which collectively suppress the expression and activity of key urea cycle enzymes, including CPS1 and OTC (De Chiara et al. 2018; Thomsen et al. 2023). Thus, exploring the overlapping mechanisms between chronological liver aging and MASLD‐associated senescence can provide critical insights into the progressive vulnerability of the hepatic urea cycle.

#### 4.1.2 Glutamine Metabolism During Aging

In tandem with the urea cycle, glutamine metabolism represents another critical arm of hepatic nitrogen handling, functioning as a high‐affinity system for ammonia clearance. This pathway is primarily regulated by two counteracting enzymes: GS, which catalyzes the ATP‐dependent synthesis of glutamine from ammonia and glutamate, and glutaminase (GLS), which hydrolyzes glutamine back into glutamate and ammonia. While the urea cycle serves as a high‐capacity conduit for bulk ammonia elimination, the exceptional affinity of GS for ammonia allows it to act as an essential scavenger. This system ensures that residual ammonia molecules are efficiently trapped before entering the systemic circulation, making the balance between glutamine synthesis and degradation vital for maintaining steady‐state nitrogen homeostasis.

During physiological aging, the regulation of glutamine metabolism undergoes complex remodeling, particularly within the context of cellular senescence. Recent breakthroughs indicate that senescent cells often experience enhanced glutaminolysis driven by the upregulation of glutaminase 1 (GLS1). For instance, it was reported that lysosomal membrane damage in senescent cells induces the expression of the kidney‐type glutaminase (KGA) isoform. The resulting localized ammonia production neutralizes intracellular acidosis, thereby promoting senescent cell survival (Johmura et al. 2021). Further illuminating this axis, recent work described a state of ‘hyperglutaminolysis’ in aged models (Chen et al. 2026), where GLS1‐driven ammonia and glutamate production fuels arginine biosynthesis, persistently activating the mTORC1 pathway to accelerate senescence. While these studies underscore significant shifts in glutamine catabolism, the senolytic potential of GLS1 inhibition remains a subject of debate, with recent cross‐laboratory studies reporting inconsistent outcomes (Kawamoto et al. 2026).

Regarding GS, research in liver disease models—which frequently mimic the metabolic insults observed in aging—suggests that this pathway is highly susceptible to hepatic stress. Studies have demonstrated that GS expression is often compromised under conditions of chronic hepatic injury or metabolic dysfunction. It is highlighted that GS is the primary enzymatic machinery for perivenous ammonia detoxification, and any functional loss in this spatially restricted zone severely impairs systemic nitrogen handling (Hakvoort et al. 2017). Furthermore, evidence was provided that GS expression is dynamically regulated in response to ammonia flux, yet this adaptive capacity is frequently impaired in damaged liver tissue (Soria et al. 2019). In addition, broader metabolic reprogramming in aged hepatocytes may alter glutamine flux, diverting glutamine toward other pathways such as energy metabolism or stress responses, thereby limiting its availability for ammonia detoxification (Chen et al. 2026; Jankowski et al. 2025). Although direct longitudinal evidence in the healthy aging liver is limited, these findings imply that the high‐affinity ammonia‐trapping capacity of GS may be particularly vulnerable to the age‐related structural and metabolic decline of the liver.

The functional synergy between the urea cycle and glutamine metabolism is structurally organized across the liver lobule, forming the classic intercellular glutamine cycle. Periportal hepatocytes, which possess the high‐capacity enzymes of the urea cycle, clear the majority of portal vein‐derived ammonia. Any residual ammonia that escapes this first barrier flows downstream to the pericentral zone, where it is sequentially captured by highly localized, high‐affinity GS. During the aging process, this precise spatial coordination and metabolic zonation face potential vulnerabilities. Age‐associated structural remodeling of hepatic sinusoids, alongside localized mitochondrial decline, may impair the efficiency of this dual‐barrier defense (Droin et al. 2021). Consequently, even a minor disruption in periportal urea synthesis can place an overwhelming metabolic burden on pericentral glutamine synthesis, demonstrating that the cooperative synergy between these two pathways is indispensable for maintaining robust hepatic ammonia handling throughout the lifespan.

### 4.2 Aging Disrupts Transcriptional and Epigenetic Control of Ammonia Metabolism

The age‐related decline in hepatic ammonia detoxification cannot be explained only by reduced enzyme abundance. Aging also perturbs transcriptional regulation, chromatin organization, and post‐translational control. These changes can weaken the coordinated expression and activity of enzymes involved in ureagenesis and glutamine‐dependent ammonia scavenging.

#### 4.2.1 Transcriptional Reprogramming in the Aging Liver

Hepatic ammonia metabolism depends on a broader transcriptional network that maintains hepatocyte identity and metabolic homeostasis. HNF4α is the best‐established regulator in this context. It controls the expression of many liver metabolic genes and is required for normal ureagenesis (Inoue et al. 2002; Odom et al. 2004). Liver‐specific loss of HNF4α impairs urea synthesis, reduces OTC expression and activity, and causes hyperammonemia (Inoue et al. 2002).

In the general hepatic context, FOXO1 is also an important metabolic regulator. It controls fasting‐responsive transcriptional programs and coordinates several aspects of hepatic nutrient metabolism (Puigserver et al. 2003; Zhang et al. 2006). C/EBPα is another key liver transcription factor. It regulates hepatic metabolic gene expression and has been implicated in the control of CPS1 expression (Chen et al. 2009; Pedersen et al. 2007). However, for FOXO1 and C/EBPα, current evidence mainly comes from studies of general liver metabolism. Direct studies linking age‐dependent changes in these factors to impaired ammonia detoxification in the aging liver are still limited.

Therefore, aging‐associated transcriptional reprogramming is likely to contribute to impaired ammonia detoxification by weakening the coordinated expression of genes required for efficient ureagenesis and ammonia scavenging.

#### 4.2.2 Epigenetic Alterations Affecting Nitrogen Metabolism Genes

Epigenomic remodeling is an important feature of liver aging, although the available evidence does not support a uniform model of globally reduced chromatin accessibility. In aged mouse liver, promoter and enhancer accessibility may increase, whereas promoter‐proximal pausing of RNA polymerase II declines, indicating that transcriptional output becomes dysregulated rather than simply repressed (Pedersen et al. 2007). In parallel, aging is associated with redistribution of repressive histone marks, including broad accumulation of H3K27me3, which contributes to a hyper‐quiescent chromatin state and suppression of liver‐function programs (Bozukova et al. 2022; Chen et al. 2009). Collectively, these findings suggest that aging reshapes chromatin organization and transcriptional competence in hepatocytes, thereby impairing the coordinated regulation of metabolic pathways, including those involved in nitrogen metabolism.

#### 4.2.3 Post‐Translational Regulation

Post‐translational regulation provides an additional layer of control over hepatic ammonia detoxification. CPS1, the rate‐limiting enzyme of the urea cycle, is activated by SIRT5‐dependent deacylation/deacetylation, and disruption of this mechanism impairs adaptive ureagenesis and promotes hyperammonemia (Abudahab et al. 2024). Because aging is broadly associated with altered NAD^+^/sirtuin signaling and changes in mitochondrial protein acylation, the efficiency of CPS1 regulation may decline in old liver (Yang et al. 2023). In addition, age‐related remodeling of hepatic phosphorylation networks has been reported, further supporting the concept that post‐translational dysregulation contributes to the deterioration of metabolic homeostasis in aging (Nakagawa et al. 2009). Together, these findings indicate that aging‐induced imbalance in post‐translational control may aggravate the decline in ammonia detoxification at the protein level.

Beyond the urea cycle, GS, which serves as the critical secondary ammonia scavenger, is also tightly controlled by post‐translational modifications, rendering it highly vulnerable to the aged hepatic microenvironment. Crucially, biochemical studies have demonstrated that the catalytic activity of mammalian GS is profoundly inhibited by tyrosine nitration under conditions of oxidative and nitrosative stress (Gorg et al. 2007). Because physiological aging and associated mitochondrial dysfunction are intrinsically coupled with elevated nitrosative and oxidative stress, an age‐dependent increase in GS nitration could significantly blunt pericentral ammonia detoxification. Furthermore, GS protein abundance is dynamically regulated through an acetylation‐dependent degradation pathway. Specifically, acetylation at distinct lysine residues (K11 and K14) creates a degron recognized by the CRL4‐CRBN ubiquitin ligase complex, marking the enzyme for proteasomal degradation (Nguyen et al. 2016). Given the broad age‐related dysregulation in hepatic acetylation networks, such as declining sirtuin availability, this acetylation‐ubiquitination crosstalk may further destabilize GS protein levels, synergistically impairing the liver's overall capacity for nitrogen clearance alongside urea cycle deficits.

### 4.3 Metabolic Zonation Changes

Beyond molecular regulation, ammonia metabolism is critically dependent on the spatial organization of metabolic functions within the liver. This spatial division of labor ensures highly efficient and robust ammonia clearance.

#### 4.3.1 Mechanisms Maintaining Hepatic Zonation

Under physiological conditions, hepatic zonation is maintained by integrated morphogen gradients and microenvironmental cues. Canonical Wnt/β‐catenin signaling plays a central role in establishing and preserving pericentral identity by promoting expression of GS and other pericentral genes, whereas periportal programs, including those related to the urea cycle, predominate where β‐catenin activity is lower (Ji et al. 2025; Mohallem et al. 2025). In addition, gradients of oxygen, nutrients, and hormones across the hepatic lobule shape metabolic compartmentalization: periportal regions favor oxidative metabolism and ureagenesis, whereas pericentral regions are specialized for glutamine synthesis and xenobiotic metabolism (Haussinger 1990). Liver sinusoidal endothelial cells further reinforce zonation by supplying Wnt ligands, particularly Wnt2 and Wnt9b, to the pericentral niche (Benhamouche et al. 2006). Recent single‐cell and spatial transcriptomic studies have confirmed that these signaling gradients generate stable and highly reproducible patterns of gene expression across the lobule (Kietzmann 2017).

#### 4.3.2 Loss of Zonation in the Aging Liver

Recent evidence indicates that aging disrupts the molecular and cellular machinery required to maintain hepatic zonation. In both mice and humans, aging is associated with blurring of periportal and pericentral hepatocyte identities, expansion of zonal domains, and the emergence of aberrant bi‐zonal hepatocytes co‐expressing markers that are normally spatially segregated (Halpern et al. 2017; Hu et al. 2022).

Mechanistically, this deterioration has been linked to altered expression of key zonation regulators, including components of the Wnt/β‐catenin pathway, together with age‐dependent changes in non‐parenchymal cell signaling (Halpern et al. 2017; Hu et al. 2022). Structural remodeling of the aged sinusoid, including capillarization, reduced fenestration, and impaired microcirculatory function, is also likely to weaken the local gradients that normally maintain metabolic specialization (Sinha et al. 2025). In addition, chronic low‐grade inflammation, senescence‐associated secretory phenotypes, and age‐related fibrogenic remodeling can further distort the lobular microenvironment and destabilize spatial patterning (Cogger et al. 2014; Maeso‐Diaz et al. 2018).

#### 4.3.3 Functional Consequences for Ammonia Metabolism

Loss of metabolic zonation is expected to have major consequences for ammonia detoxification. Physiologically, ammonia is first converted to urea in periportal hepatocytes, and residual ammonia is subsequently scavenged by pericentral GS. When this sequential architecture is disrupted, the overall efficiency of hepatic ammonia clearance is reduced (Le Couteur et al. 2025). This concept is supported by genetic studies showing that perturbation of β‐catenin‐dependent zonation alters the spatial distribution of periportal urea‐cycle genes and pericentral GS, thereby impairing hepatic ammonia metabolism (Karpova et al. 2026). In the context of aging, the recently described mixing and expansion of zonal programs strongly suggest that the normal handoff between ureagenesis and GS‐dependent ammonia scavenging becomes less precise (Halpern et al. 2017; Le Couteur et al. 2025). Because hyperammonemia itself can induce hepatocellular stress, including mitochondrial dysfunction and oxidative injury, impaired zonation may create a vicious cycle in which defective detoxification further aggravates liver dysfunction (Gallego‐Duran et al. 2024; Kerbert et al. 2025).

## 5 Molecular Mechanisms Linking Ammonia Metabolism and Liver Aging

Aging weakens the transcriptional control, epigenetic stability, and metabolic zonation that support efficient ammonia detoxification. In turn, impaired ammonia handling may further accelerate liver aging. Current evidence suggests that ammonia is not only a toxic by‐product of nitrogen metabolism but also a stressor that perturbs mitochondrial homeostasis, senescence‐related signaling, proteostasis, and the hepatic tissue niche. These processes may together promote the structural and functional decline of the aging liver.

### 5.1 Mitochondrial Dysfunction, Oxidative Stress, and Bioenergetic Failure

Mitochondria are central to hepatic ammonia detoxification because the first steps of the urea cycle occur in the mitochondrial matrix. Aging is accompanied by reduced mitochondrial fitness in the liver, including impaired oxidative metabolism and declining organelle quality control (Le Couteur et al. 2025). Ammonia can further aggravate this vulnerability. It disrupts mitochondrial metabolism, alters redox balance, and promotes reactive oxygen species generation. It can also impair membrane potential and amplify oxidative stress, which further weakens mitochondrial performance (Gallego‐Duran et al. 2024; Moedas et al. 2024).

These changes are important because ureagenesis is an ATP‐demanding process. Once mitochondrial ATP production falls, ammonia detoxification becomes less efficient. This may create a self‐reinforcing cycle. Hyperammonemia impairs mitochondrial function and increases oxidative stress, whereas reduced mitochondrial performance lowers the energy supply needed for effective ureagenesis (Bigot et al. 2017; Zong et al. 2024). In experimental models, enhancement of hepatic autophagy improves ureagenesis and protects against hyperammonemia, which further supports the close link between mitochondrial quality control, energy balance, and ammonia disposal (Soria et al. 2018). Thus, ammonia‐associated mitochondrial stress is a plausible mechanism by which metabolic dysfunction can accelerate hepatocellular aging.

### 5.2 Genomic Instability and the Senescence Cascade

Persistent ammonia stress may also promote senescence‐associated signaling. In general senescence biology, oxidative and metabolic stress can activate the DNA damage response and induce p53/p21‐dependent growth arrest. In hyperammonemic extrahepatic models, ammonia has been shown to increase p53 and p21 expression and to induce senescence‐like phenotypes. This has been demonstrated in skeletal muscle cells and, more recently, in brain endothelial cells (Kumar et al. 2021; Orzel‐Gajowik et al. 2025). These observations support the idea that chronic ammonia stress can favor the transition from reversible injury to stable senescence outside the liver.

In the liver, senescence is strongly linked to p53/p21 signaling and to acquisition of the SASP (Almalki and Almujri 2024). Therefore, it is reasonable to propose that sustained ammonia‐associated oxidative and metabolic stress may amplify senescence pathways in hepatocytes and non‐parenchymal liver cells. Such a response could reduce functional hepatocyte mass and strengthen the low‐grade inflammatory milieu that characterizes liver aging (Li et al. 2023). However, direct evidence showing that ammonia specifically drives a complete p53/p21‐SASP program in aged hepatocytes remains limited. This point should therefore be presented cautiously.

### 5.3 Metabolic–Epigenetic Coupling Through α‐Ketoglutarate

α‐Ketoglutarate (α‐KG) is a tricarboxylic acid cycle intermediate. It is also a key metabolic cofactor linked to cellular plasticity and aging biology (Naeini et al. 2023; Schvartzman et al. 2018; Soria and Brunetti‐Pierri 2019). During hyperammonemia, α‐KG can be consumed during ammonia fixation and glutamate synthesis, and hepatic α‐KG depletion has been documented in experimental models (Soria et al. 2018; Soria and Brunetti‐Pierri 2019).

This metabolic shift is relevant because α‐KG is required by several 2‐oxoglutarate‐dependent dioxygenases, including TET DNA demethylases and Jumonji‐domain histone demethylases (Schvartzman et al. 2018). Reduced α‐KG availability may therefore limit demethylase activity and favor a more repressive chromatin state. Because α‐KG availability has been broadly linked to cell fate control and senescence‐related biology, ammonia‐driven α‐KG depletion may help stabilize dysfunctional transcriptional programs in aging liver cells (Naeini et al. 2023). At present, however, direct evidence that ammonia causes locus‐specific DNA or histone hypermethylation in aged hepatocytes is still limited. This mechanism is therefore best described as biologically plausible rather than fully established.

### 5.4 Loss of Proteostasis: Autophagy Defect and Impaired Protein Homeostasis

Maintenance of proteostasis is essential for hepatocyte longevity. Ammonia can disturb this system at the level of the lysosome. Because ammonia readily accumulates in acidic organelles as ammonium, it can compromise lysosomal homeostasis and impair lysosome‐dependent proteolysis. This effect is particularly important in the liver, where autophagy supports ammonia homeostasis, ureagenesis, and mitochondrial quality control (Levin‐Konigsberg et al. 2025).

Ammonia may also affect protein synthesis. In extrahepatic hyperammonemic models, especially skeletal muscle, ammonia suppresses anabolic signaling and reduces protein synthesis through mechanisms involving eIF2α phosphorylation and impaired mTORC1 signaling (Davuluri et al. 2016; Kumar et al. 2017). These findings suggest that ammonia can disturb both arms of proteostasis, namely protein synthesis and protein turnover. In the liver, direct evidence for the same translational mechanism in aged hepatocytes is still limited. Even so, impaired lysosomal degradation together with reduced biosynthetic capacity would be expected to intensify proteotoxic stress and lower cellular resilience during aging.

### 5.5 Ammonia‐Driven Inflammatory and Fibrogenic Niche Remodeling

Ammonia may also promote liver aging by reshaping the inflammatory microenvironment. In addition to its direct effects on hepatocytes, ammonia can act on non‐parenchymal liver cells. A recent review highlighted inflammation as one of the major downstream stress responses triggered by ammonia in liver disease (Gallego‐Duran et al. 2024). In cultured human hepatic stellate cells, pathophysiological ammonia concentrations increase reactive oxygen species, alter cell morphology, enhance contractility, and induce pro‐inflammatory gene expression together with activation markers such as α‐SMA and PDGFRβ (Jalan et al. 2016). These findings indicate that ammonia can directly prime a more reactive and inflammatory cellular niche within the liver.

Ammonia also appears to promote fibrogenic remodeling. In experimental steatotic liver disease, ammonia scavenging attenuates fibrosis progression, which supports a causal contribution of ammonia to liver fibrogenesis (De Chiara et al. 2020). More recent in vivo work further shows that chronic hyperammonemia can induce liver fibrogenesis and programmed liver cell death even in the absence of pre‐existing liver disease (Kerbert et al. 2025). Together, these studies suggest that ammonia is not merely a marker of impaired detoxification. It can also function as an active mediator of tissue remodeling. In the aging liver, where low‐grade inflammation and maladaptive repair are already present, ammonia may amplify crosstalk among hepatocytes, stellate cells, endothelial cells, and immune cells, thereby accelerating niche dysfunction.

**FIGURE 2:**
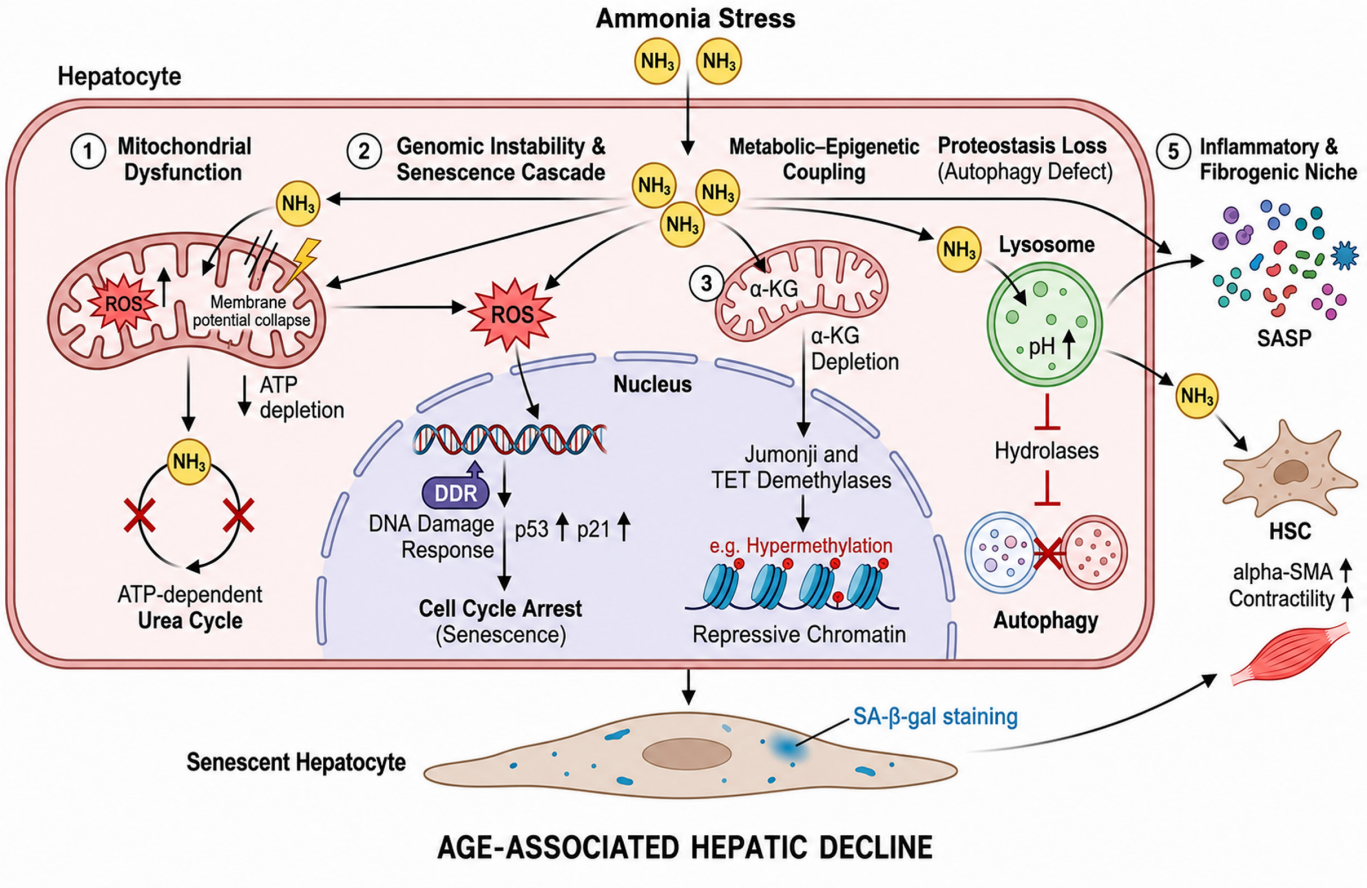
Molecular mechanisms linking ammonia stress and liver aging. Elevated intracellular ammonia disrupts multiple vital cellular processes in aged hepatocytes. (1) Ammonia induces mitochondrial dysfunction, characterized by reactive oxygen species (ROS) overproduction and ATP depletion. (2) This metabolic stress triggers genomic instability and a senescence cascade via the DNA damage response (DDR) and p53/p21 activation. (3) Ammonia‐driven depletion of α‐ketoglutarate (α‐KG) promotes repressive epigenetic remodeling. (4) Accumulation of ammonia impairs lysosomal acidification, leading to defective autophagy and proteostasis loss. (5) Ultimately, senescent hepatocytes develop a senescence‐associated secretory phenotype (SASP), which activates adjacent hepatic stellate cells (HSCs) and promotes an inflammatory and fibrogenic microenvironment.

Taken together, current evidence supports a bidirectional relationship between ammonia dysregulation and liver aging. Aging reduces the efficiency and spatial precision of ammonia detoxification, whereas excess ammonia can further impair mitochondrial function, reinforce senescence‐related stress programs, disturb proteostasis, and remodel the hepatic microenvironment (Figure 2). Although several steps in this model still require direct validation in aged hepatocytes, the overall framework supports the view that ammonia is not only a consequence of liver dysfunction but also a potential driver of age‐associated hepatic decline. This concept also provides a natural transition to the next section, which addresses the role of ammonia in cross‐organ communication and extrahepatic dysfunction.

## 6 Systemic Effects of Ammonia Dysregulation in Aging

Aging impairs the hepatic networks that support efficient ammonia detoxification, whereas excess ammonia can further aggravate liver dysfunction. The consequences of this imbalance are not restricted to the liver. Because the liver is the central organ for ammonia clearance, impaired ammonia homeostasis can influence the function of distant tissues and contribute to systemic decline during aging. Current evidence highlights three major axes of interaction, namely the liver–brain, liver–muscle, and liver–gut axes.

### 6.1 The Liver–Brain Axis: Cognitive Decline and Neuroinflammation

The brain is highly sensitive to changes in circulating ammonia. When hepatic detoxification declines, ammonia can enter the brain and is handled mainly by astrocytes through glutamine synthetase. Because astrocytes do not possess a complete urea cycle, this pathway leads to intracellular glutamine accumulation. Excess glutamine acts as an osmolyte and contributes to astrocyte swelling, which is a well‐recognized feature of hyperammonemia‐related brain injury (Brusilow et al. 2010; Haussinger et al. 2022).

Ammonia also perturbs several processes that are essential for normal brain function. In experimental and clinical studies of hepatic encephalopathy, hyperammonemia has been linked to mitochondrial dysfunction, oxidative stress, and impaired neurotransmitter balance in the central nervous system (Rodrigo et al. 2010). These changes can interfere with synaptic transmission and reduce the efficiency of neuronal communication. Ammonia is also associated with activation of microglia and increased production of inflammatory mediators, which further amplifies neuronal stress and disrupts brain homeostasis (Rodrigo et al. 2010).

These effects are especially relevant in aging. Older individuals generally have lower neural reserve and greater susceptibility to chronic inflammation. Under these conditions, even modest but sustained elevations in ammonia may have clinically meaningful consequences. Minimal hepatic encephalopathy is one example of this process. It is characterized by subtle deficits in attention, psychomotor speed, working memory, and executive function (Weissenborn 2019). Although these abnormalities are often subclinical, they can significantly affect daily functioning and quality of life. Taken together, the liver–brain axis provides an important framework for understanding how impaired ammonia handling may contribute to cognitive vulnerability during aging.

### 6.2 The Liver–Muscle Axis: Sarcopenia and Metabolic Failure

Skeletal muscle is an important extrahepatic site of ammonia disposal. When hepatic ammonia clearance is impaired, muscle helps buffer excess ammonia through glutamine synthesis. This compensatory role becomes particularly important in chronic liver dysfunction and may partially offset reduced hepatic detoxification capacity (Jindal and Jagdish 2019). However, prolonged exposure to high ammonia concentrations also has detrimental effects on muscle itself.

Experimental studies have shown that ammonia enters skeletal muscle through Rh glycoproteins and activates NF‐κB‐dependent myostatin signaling, thereby suppressing muscle growth and promoting muscle wasting (Davuluri et al. 2016; Qiu et al. 2013). Hyperammonemia also impairs muscle protein homeostasis. It suppresses anabolic signaling, reduces protein synthesis, and disrupts mitochondrial function, which together contribute to anabolic resistance and progressive loss of muscle mass (Kumar et al. 2017; Qiu et al. 2013). In this setting, muscle is no longer simply a compensatory organ. It gradually becomes another target of ammonia toxicity.

This interaction is particularly important in the context of aging because sarcopenia is already a common feature of later life. Older individuals have reduced muscle reserve, lower regenerative capacity, and diminished resilience to metabolic stress. Ammonia burden may therefore accelerate pre‐existing age‐related muscle decline. At the same time, loss of muscle mass reduces the body's capacity to buffer ammonia, thereby creating a maladaptive feedback loop between liver dysfunction and muscle wasting (Jindal and Jagdish 2019). This reciprocal relationship links hyperammonemia not only to sarcopenia, but also to frailty, weakness, and reduced physiological reserve. For this reason, the liver–muscle axis is increasingly recognized as a clinically relevant component of systemic aging.

### 6.3 The Liver–Gut Axis: Microbiota Dysbiosis and Intestinal Barrier Dysfunction

The gut is a major source of systemic ammonia. Intestinal bacteria generate ammonia through urease activity and through deamination of nitrogen‐containing substrates. The amount of ammonia reaching the portal circulation therefore depends on both microbial metabolism and intestinal barrier function (Jakhar et al. 2024). Under physiological conditions, this ammonia load is efficiently cleared by the liver. However, once hepatic detoxification becomes compromised, gut‐derived ammonia can make a larger contribution to systemic exposure.

Aging is associated with broad remodeling of the gut ecosystem. These changes include reduced microbial diversity, altered community composition, and impaired intestinal homeostasis (Claesson et al. 2012; Thevaranjan et al. 2017). Dysbiosis may shift microbial metabolism toward greater ammonia production, particularly when urease‐positive or proteolytic microbial populations become enriched (Jakhar et al. 2024). At the same time, aging is often accompanied by increased intestinal permeability and a tendency toward low‐grade mucosal inflammation (Claesson et al. 2012; Thevaranjan et al. 2017). These barrier defects allow not only ammonia, but also microbial products, to enter the portal circulation more readily.

The resulting signals can intensify hepatic stress and may further destabilize ammonia homeostasis. In addition, liver dysfunction can feed back to the intestine through altered bile acid metabolism, nutrient flux, and host–microbial signaling. This creates a bidirectional cycle in which gut dysbiosis worsens hepatic dysfunction, and hepatic dysfunction further reshapes the intestinal environment. In aging, such a cycle may be especially important because both liver resilience and intestinal barrier integrity are already diminished. For this reason, the liver–gut axis is increasingly viewed as a key target for strategies aimed at lowering systemic ammonia burden and preserving metabolic stability in later life.

**FIGURE 3:**
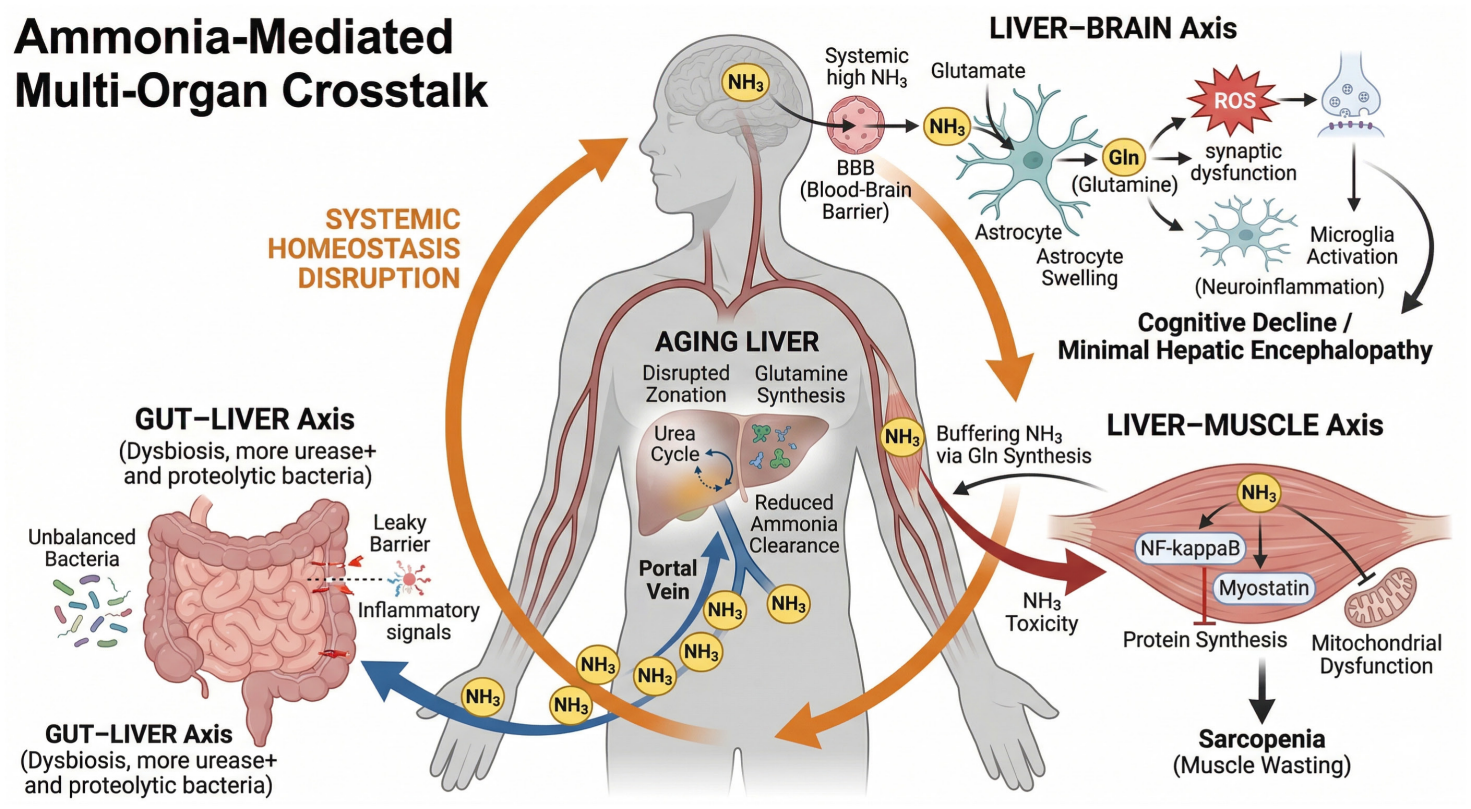
Ammonia‐mediated multi‐organ crosstalk. Impaired hepatic ammonia clearance in the aging liver disrupts systemic homeostasis through interconnected multi‐organ axes. In the Liver‐Brain Axis, circulating ammonia crosses the blood–brain barrier, leading to glutamine (Gln) accumulation, astrocyte swelling, and microglia activation, ultimately contributing to cognitive decline and minimal hepatic encephalopathy. In the Liver‐Muscle Axis, skeletal muscle initially buffers ammonia via Gln synthesis; however, persistent ammonia toxicity inhibits protein synthesis via NF‐κB and myostatin activation, accelerating sarcopenia. In the Gut‐Liver Axis, age‐associated microbiota dysbiosis (enrichment of urease‐positive bacteria) and a leaky intestinal barrier exacerbate the portal ammonia burden and inflammatory signaling, creating a vicious cycle that further deteriorates liver function.

Overall, hyperammonemia should be viewed as a systemic disturbance rather than an isolated defect of hepatic nitrogen metabolism. Because the liver is the principal site of ammonia detoxification, age‐related impairment of hepatic ammonia handling may serve as an initiating event that gradually influences other organs through sustained elevation of circulating ammonia (Figure 3). The brain, skeletal muscle, and gut currently represent the best‐characterized targets of this process. However, whether ammonia dysregulation also affects additional organs and tissues during aging remains unclear and warrants further investigation. Clarifying these systemic pathways will help refine our understanding of ammonia‐related organ crosstalk and may also support the development of more effective intervention strategies.

## 7 Therapeutic and Translational Perspectives

While significant clinical progress has been made in managing pathological hyperammonemia, it is imperative to acknowledge a major gap in the current literature: established therapeutic interventions for ammonia dysregulation currently lack robust experimental and clinical support in the specific context of physiological liver aging. The pharmacological agents and dietary strategies discussed herein have predominantly been validated in severe disease models, such as hepatic encephalopathy, acute liver failure, or chronic metabolic disorders. Therefore, this section aims to systematically summarize existing therapeutic paradigms derived from these hepatic pathologies. By consolidating these strategies, we seek to provide a conceptual framework and outline prospective translational avenues, highlighting how targeted modulation of ammonia metabolism might be systematically investigated in the future to safeguard hepatic resilience and mitigate nitrogen‐related vulnerabilities throughout the lifespan.

### 7.1 Conventional and Repurposed Ammonia‐Lowering Agents

Current pharmacological treatment of hyperammonemia mainly aims to reduce intestinal ammonia production or enhance ammonia disposal. In clinical practice, lactulose and rifaximin remain the best‐established gut‐directed therapies, especially in the context of hepatic encephalopathy. These agents lower the intestinal ammonia burden by altering luminal pH, reducing ammonia absorption, and modulating the gut microbiota (Fu et al. 2022; Moon et al. 2023). Although these treatments are strictly established for pathological liver diseases, they offer a conceptual framework for lowering chronic ammonia stress, provided their long‐term safety and efficacy in the context of physiological aging are rigorously validated.

L‐ornithine L‐aspartate (LOLA) is another important ammonia‐lowering agent. It provides substrates that support urea synthesis in residual periportal hepatocytes and glutamine synthesis in perivenous hepatocytes and skeletal muscle (Butterworth et al. 2018). Extrapolating from these therapeutic outcomes in chronic liver diseases, LOLA may be theoretical relevant when age‐related metabolic decline reduces the efficiency of endogenous ammonia detoxification. However, most current evidence comes from studies in cirrhosis and hepatic encephalopathy rather than physiological liver aging (Sidhu et al. 2018). Its long‐term value in older individuals with subclinical ammonia dysregulation therefore remains to be established.

Branched‐chain amino acids (BCAAs), especially leucine‐enriched formulations, may also have translational value. In hyperammonemic states, leucine has been shown to improve skeletal muscle proteostasis and to partially counteract ammonia‐associated anabolic resistance (Kumar et al. 2017). Because skeletal muscle serves as an important extrahepatic site of ammonia buffering, BCAA supplementation may support the liver–muscle axis while also helping preserve muscle mass. Even so, whether this strategy can directly improve ammonia handling in physiological aging populations without advanced liver disease remains uncertain and requires further study.

### 7.2 Targeting Mitochondrial Health and NAD ^+^ Homeostasis

Mitochondrial dysfunction is a plausible therapeutic target because the first steps of the urea cycle occur in the mitochondrial matrix and because ammonia‐associated stress can further impair mitochondrial performance. One mechanistically attractive approach is restoration of NAD^+^‐dependent signaling. CPS1, the rate‐limiting enzyme of the urea cycle, is regulated by the NAD^+^‐dependent deacylase SIRT5, which promotes CPS1 activity and supports nitrogen disposal (Nakagawa et al. 2009). More recent work also suggests that NAD^+^ supplementation can enhance oxidative metabolism and nitrogen elimination through a SIRT5‐dependent mechanism (Richard et al. 2026). These findings provide a rationale for exploring NAD^+^ precursors as metabolic interventions in settings of impaired ammonia clearance.

Additional approaches aimed at improving mitochondrial quality control may also be relevant. Agents that enhance mitophagy or reduce mitochondrial oxidative stress could, in principle, preserve the bioenergetic capacity required for effective ureagenesis. Urolithin A is one example that has attracted interest because of its mitophagy‐promoting and geroprotective properties in preclinical and early clinical studies (Liu et al. 2022). At present, however, direct evidence that such agents improve ammonia detoxification in the context of chronological liver aging is still lacking. Their therapeutic role should therefore be considered promising but preliminary.

### 7.3 Senotherapeutics: A Potential Strategy for Preserving Hepatic Resilience

Because senescent cells accumulate in the aging liver and contribute to chronic inflammation, metabolic dysfunction, and niche remodeling, senotherapeutics have emerged as a conceptually attractive strategy. This class includes senolytics, which selectively eliminate senescent cells, and senomorphics, which suppress harmful features of the senescent phenotype without necessarily killing the cells (Du et al. 2026). In principle, reducing senescent‐cell burden could relieve local inflammatory pressure, improve tissue homeostasis, and indirectly support metabolic pathways involved in ammonia handling.

While senotherapeutics have demonstrated efficacy in specific preclinical disease models, such as diet‐induced steatosis, recent liver‐focused reviews emphasize that the efficacy and safety of senotherapeutic interventions depend strongly on cell type, disease context, and drug specificity (Du et al. 2026). Likewise, newer experimental work suggests that not all commonly used senolytics are effective across liver disease models, and some agents may even have context‐dependent adverse effects (Du et al. 2026). For this reason, it would be premature to conclude that senotherapeutics can already restore hepatic zonation or normalize ammonia metabolism. A more appropriate view is that while senescence‐targeted therapy holds conceptual promise, it must first be determined whether eliminating senescent cells specifically rescues ureagenesis or glutamine synthesis in physiological aging models before considering its translational potential.

### 7.4 Precision Microbiota Modulation and Barrier Repair

Given the central role of the liver–gut axis in systemic ammonia balance, microbiota‐directed therapy represents another major translational avenue. Probiotics, prebiotics, lactulose, and rifaximin can all shift intestinal metabolism away from excessive ammonia production, although the strength of evidence differs among these interventions (Bloom et al. 2021). In patients with hepatic encephalopathy, microbiota‐directed treatment can improve cognitive outcomes and lower ammonia‐related toxicity, which supports the broader idea that gut‐derived ammonia is a modifiable therapeutic target (Bloom et al. 2021).

More intensive approaches, including fecal microbiota transplantation (FMT), are also being explored. Early‐phase clinical and translational studies suggest that FMT may improve outcomes in hepatic encephalopathy by restoring microbial diversity, reducing the abundance of urease‐associated dysbiosis, and improving gut–liver–brain signaling (Bloom et al. 2021). In parallel, strategies that strengthen intestinal barrier function may help reduce portal exposure to both ammonia and other gut‐derived inflammatory signals (Tandon et al. 2017).

These findings in severe liver diseases raise the intriguing possibility that similar combined approaches may be attractive in the context of aging, as they target both the source of ammonia and one of the major amplifiers of chronic hepatic stress. Ultimately, whether these established interventions yield comparable efficacy in preserving physiological nitrogen homeostasis throughout the lifespan remains a critical question for future clinical evaluation.

## 8 Conclusions

Ammonia metabolism is a central but still underappreciated component of liver aging. Although severe hyperammonemia is classically associated with advanced liver disease, the evidence summarized in this review suggests that even milder disturbances in ammonia handling may be relevant in physiological aging. Aging affects multiple layers of hepatic ammonia detoxification, including mitochondrial function, enzyme regulation, transcriptional and epigenetic control, and metabolic zonation. These changes are likely to reduce the efficiency and precision of nitrogen disposal, even when overt liver failure is absent.

At the same time, ammonia should no longer be viewed solely as a passive metabolic waste product. Current evidence indicates that excess ammonia can act as a biologically active stressor that perturbs mitochondrial homeostasis, promotes oxidative and senescence‐related stress, impairs proteostasis, and reshapes the inflammatory and fibrogenic liver niche. In this way, impaired ammonia detoxification may not only result from liver aging but may also feed back to accelerate it. This bidirectional model provides a useful framework for understanding how metabolic decline becomes progressively amplified in the aging liver.

An important implication of this concept is that ammonia dysregulation is not restricted to the liver itself. Once hepatic clearance becomes inefficient, elevated ammonia may influence the brain, skeletal muscle, and gut, thereby contributing to cognitive vulnerability, sarcopenia, chronic inflammation, and broader systemic decline. These interorgan effects strengthen the idea that ammonia imbalance should be considered a systemic disturbance initiated by dysfunction in a key metabolic organ.

Several questions remain unresolved. Direct evidence for impaired ammonia handling during normal aging is still limited, and many proposed mechanisms have not yet been fully validated in aged hepatocytes or in physiologically aged animal models. In particular, future studies should clarify how aging alters the balance between ureagenesis and glutamine synthesis, how zonation loss affects ammonia clearance in vivo, and whether ammonia directly drives senescence and tissue remodeling in the aging liver. It will also be important to determine whether additional organs are involved in ammonia‐related interorgan communication during aging.

From a translational perspective, the pathways discussed here suggest several promising therapeutic directions. Conventional ammonia‐lowering therapies, metabolic interventions that support mitochondrial and nicotinamide adenine dinucleotide‐dependent function, senescence‐targeted strategies, and microbiota‐directed approaches may all contribute to restoring ammonia homeostasis. Crucially, the translational scope of targeting age‐related ammonia dysregulation expands significantly when considering MASLD. Since cellular senescence acts as a shared executioner in both chronological liver aging and MASLD progression, therapeutic strategies aimed at modulating hepatic ammonia pathways—such as utilizing senolytic agents to suppress hyperglutaminolysis or optimizing nitrogen scavengers—hold profound clinical promise. Future clinical trials should therefore position MASLD, a highly common chronic liver disease, as a pivotal human model to evaluate whether mitigating senescence‐driven ammonia accumulation can successfully ameliorate chronic hepatic decline. A more complete mechanistic understanding of ammonia dysregulation in aging may therefore not only improve our knowledge of liver senescence but also open new opportunities to preserve metabolic health and reduce multi‐organ vulnerability in later life.

## Author Contributions

H.Z.: writing – original draft preparation and visualization. G.L.: writing – review and editing. A.L.: conceptualization and funding acquisition.

## Funding

This work was supported by the National Natural Science Foundation of China (No. 82470657).

## Conflicts of Interest

The authors declare no conflicts of interest.

## Data Availability

Data sharing not applicable to this article as no datasets were generated or analysed during the current study.
